# Psychosocial occupational health in military work environments: cross-sectional findings among Spanish enlisted military personnel

**DOI:** 10.3389/fpubh.2026.1996254

**Published:** 2026-10-08

**Authors:** José Javier Miranda Mayo, María M. Fernández-Valera, María Concepción Pérez-Cárceles

**Affiliations:** 1 Universidad Politécnica de Cartagena (UPCT), Cartagena, Spain; 2 Centro Universitario de la Defensa de San Javier (affiliated to Universidad Politécnica de Cartagena), San Javier, Spain

**Keywords:** job stress, military identity, occupational health, occupational health psychology, psychological capital, psychosocial risk, work engagement

## Abstract

Military work environments combine high operational demands, hierarchical control and limited autonomy, creating conditions in which psychosocial risks may affect occupational health. This study examined an integrated psychosocial occupational health model linking job stress, distributive justice, psychological capital, military identity and occupational health outcomes among Spanish enlisted military personnel. A cross-sectional online survey was conducted with 382 enlisted members of the Spanish Armed Forces between February 17 and March 30, 2025. Measures assessed job stress, distributive justice, psychological capital, military identity, well-being, job satisfaction and work engagement. Direct and indirect associations were estimated using partial least squares structural equation modelling with 10,000 bootstrap samples. Job stress was negatively associated with psychological capital, distributive justice, military identity and occupational health outcomes. Psychological capital and military identity were positively associated with occupational health outcomes, and the model explained 73.5% of their variance. Indirect effects indicated that job stress was associated with poorer occupational health mainly through lower psychological capital and, to a lesser extent, lower military identity. The findings suggest that occupational health in military work environments is associated not only with job stress but also with personal, organisational and identity-related resources. Psychosocial risk prevention in military and other hierarchical workplaces may benefit from combining stressor reduction with strategies that strengthen psychological capital, distributive fairness and constructive occupational identity.

## Introduction

Military work environments (e.g., armed forces and other highly regimented services) expose personnel to sustained operational demands, irregular schedules, deployment cycles, and high-consequence safety requirements, alongside hierarchical control and role-based constraints (1, 2). These characteristics may be associated with heightened psychosocial risks and poorer occupational health, particularly among enlisted personnel who typically have limited decision latitude and fewer organisational resources.

Occupational health has traditionally been defined as a multidimensional construct encompassing workers’ physical, psychological, and social well-being. Contemporary approaches increasingly emphasise the importance of psychosocial working conditions, recognising that employee health is associated not only with exposure to physical hazards but also with organisational practices, interpersonal relationships, perceived fairness, and access to personal and organisational resources. In highly demanding occupations, the balance between occupational demands and available resources has become a central concern for understanding employee well-being, work engagement, and long-term organisational sustainability.

Military organisations constitute a particularly relevant context for examining these processes. Unlike most civilian workplaces, military institutions are characterised by formal hierarchies, strong normative regulation, restricted autonomy, collective responsibility, and an organisational culture emphasising discipline, cohesion, and commitment to institutional goals. Personnel are often required to perform under conditions of uncertainty, operational pressure, geographical mobility, and potential exposure to danger. These characteristics create a work environment in which psychosocial demands and resource-related processes may be especially salient.

Within military organisations, enlisted personnel represent a subgroup of particular interest. In addition to operational demands common to military service, enlisted personnel frequently occupy lower hierarchical positions and may experience reduced influence over organisational decisions affecting their careers and working conditions. In the Spanish Armed Forces, these challenges are further intensified by a distinctive career structure in which a substantial proportion of enlisted personnel must leave active service at the age of 45 unless they obtain permanent status. This institutional characteristic introduces an additional source of uncertainty regarding future career opportunities and long-term occupational stability. Consequently, understanding the psychosocial mechanisms associated with occupational health in this population is not only relevant from an employee well-being perspective but also from an organisational effectiveness and workforce sustainability perspective.

Although military occupational health has received increasing attention in recent decades, research has predominantly focused on psychopathology, operational stress, trauma exposure, post-traumatic stress disorder, and mental health disorders. Comparatively less attention has been devoted to examining how personal, organisational and identity-related resources are jointly associated with positive occupational health outcomes in military populations. This limitation is important because contemporary occupational health theories suggest that employee well-being is not merely the absence of psychological distress but also the presence of positive psychological functioning, motivation, and adaptive organisational experiences.

Conservation of resources theory proposes that stress may arise when valued resources are threatened, lost, or insufficiently gained after investment, and that resource losses may be involved in loss spirals under chronic demands (3). The Job Demands–Resources (JD-R) model similarly suggests that high demands are associated with energy depletion and poorer well-being, whereas personal and organisational resources are associated with motivation and health (4–6). Effort-reward imbalance theory further emphasises the health costs of high effort coupled with low reward and low recognition (7). Together, these perspectives suggest that psychosocial stressors in military settings may be associated with poorer occupational health both directly and through resource-related pathways. Recent extensions of the JD-R model have also highlighted the value of incorporating organisational justice into demand–resource frameworks when examining work attitudes over time (8), which is particularly relevant in hierarchical contexts where recognition, career opportunities and perceived fairness are strongly shaped by formal organisational structures.

These theoretical frameworks provide a strong foundation for understanding occupational health in military settings. However, they do not explicitly address certain characteristics that may be particularly relevant in military organisations, including the role of military identity and the interaction between organisational fairness perceptions and identity-based processes. Consequently, there is value in examining how established occupational health theories can be integrated within a context-specific framework capable of capturing the psychosocial realities of military service.

The present study is grounded primarily in Adler and Castro’s Occupational Mental Health Model for the Military, which conceptualises military mental health as the result of interactions between occupational demands, personal resources, occupational resources, and health-related outcomes. The Psychosocial Occupational Health Model for Military Work Environments (MOSALPEM, from the Spanish Modelo de Salud Laboral Psicosocial para Entornos Militarizados) was developed as an operationalisable extension of this framework for the Spanish military context. Specifically, job stress was selected as an indicator of occupational demands, psychological capital as a personal resource, distributive justice as an organisational resource, and military identity as an occupation-specific resource reflecting professional identification with the military institution. Together, these constructs allow empirical testing of core mechanisms proposed by Adler and Castro while integrating complementary insights from JD-R, COR, and effort–reward imbalance theories.

Distributive justice is especially salient in hierarchical organisations because rewards (recognition, assignments, training opportunities, and career progression) are strongly shaped by rank and formal procedures. Organisational justice theory and meta-analytic evidence indicate that unfairness is associated with poorer attitudes and worse health-related outcomes (9, 10). Distributive justice captures perceived fairness of outcome allocations and is grounded in equity theory, which highlights comparisons between personal contributions and received outcomes (11, 12). In military organisations, where promotion systems, assignments and professional opportunities are highly structured, perceptions of distributive justice may be particularly relevant to employees’ attitudes towards the institution and their occupational experiences.

Personal resources have been theorised as factors that may be associated with lower vulnerability to stress-related outcomes. Psychological capital is a malleable resource comprising hope, efficacy, resilience, and optimism, and has been linked to better coping and reduced stress-related outcomes (13, 14). Within COR theory, psychological capital can be conceptualised as a strategic reservoir of personal resources that may be associated with maintained functioning despite adversity and more favourable recovery from stressful experiences. Evidence across occupational settings suggests that psychological capital is positively associated with employee well-being, job satisfaction and work engagement.

Identity-related resources may also be central in military environments. Military identity reflects the internalisation of the military role as a core component of the self and a sense of belonging to the institution (15). Stronger identification has been associated with more favourable occupational functioning, partly through social support and collective efficacy pathways in organisational contexts (16). Military identity may therefore be interpreted as a psychosocial resource associated with how personnel interpret organisational experiences, maintain commitment and report well-being in the presence of occupational stressors.

Drawing on these perspectives, we propose MOSALPEM as a context-specific model for military work environments. The model integrates key assumptions from resource-based occupational health theories while incorporating identity-related processes that may be especially relevant in military organisations. Specifically, MOSALPEM conceptualises job stress as a central occupational demand expected to be negatively associated with distributive justice, psychological capital and military identity. In turn, distributive justice is expected to be positively associated with military identity, while psychological capital and military identity are conceptualised as psychosocial resources expected to be positively associated with occupational health outcomes.

Occupational health outcomes were operationalised as a multidimensional construct comprising psychological well-being, job satisfaction, and work engagement (17–19). Although these constructs are conceptually distinct, they collectively represent complementary indicators of positive occupational functioning and adaptation. Together, they capture affective, motivational, and evaluative dimensions of employees’ experiences at work and provide a broader representation of occupational health than any individual indicator considered in isolation.

The aim of this study was to test the proposed MOSALPEM framework in a sample of enlisted personnel from the Spanish Armed Forces. Specifically, we examined the direct and indirect associations between job stress and occupational health outcomes and evaluated the mediating roles of distributive justice, psychological capital and military identity. By doing so, this study seeks to contribute to the occupational health literature by providing empirical evidence regarding the associations through which personal, organisational and identity-related resources may be linked to employee health within military work environments.

The proposed theoretical model is shown in Figure 1. In this model, JS is expected to be negatively associated with DJ, PsyCap, MI and OHO. DJ is expected to be positively associated with MI, while PsyCap and MI are expected to be positively associated with OHO. The model therefore allows the examination of both direct and indirect associations between job stress and occupational health outcomes through organisational, personal and identity-related resources.

**Figure 1:**
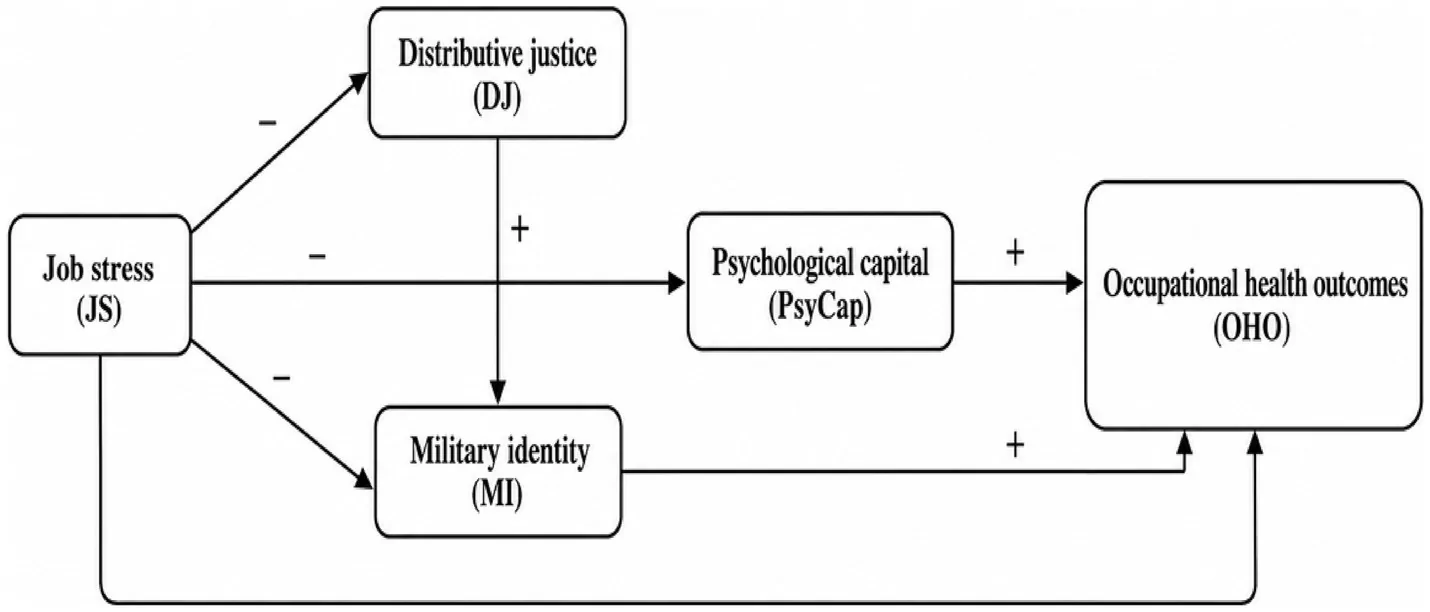
Proposed psychosocial occupational health model for military work environments (MOSALPEM). The model represents the hypothesised associations between job stress (JS), distributive justice (DJ), psychological capital (PsyCap), military identity (MI) and occupational health outcomes (OHO).

## Materials and methods

Study design and participants. This observational, cross-sectional study used an anonymous online questionnaire implemented in a purpose-built web application configured to avoid IP logging and other identifying traces. Data were collected between February 17 and March 30, 2025. Recruitment was conducted through professional military associations that disseminated the survey link via internal communication channels. By March 20, responses had largely plateaued (*n* = 353). A final phase of directed email invitations to enlisted soldiers and sailors, stratified by rank (soldier/sailor), sex, and service branch, was used to reach the target sample and better approximate the population distribution; 22 of the last 29 responses were obtained via these invitations. Inclusion criteria were active service and enlisted rank. A total of 382 complete questionnaires were analysed. Because dissemination occurred through third-party channels, the response rate cannot be precisely estimated; assuming approximately 6,900 members across collaborating associations as a maximum denominator yields a conservative minimum response rate of about 5.5% (382/6,900).

This study is reported in accordance with the STROBE statement for cross-sectional studies; a completed checklist is provided as Supplementary material.

Ethics. The study protocol was reviewed and approved by the Research Ethics Committee of the Universidad Politécnica de Cartagena, Spain, at its meeting of 18 December 2024, for the R&D&i activity entitled “Salud laboral en el contexto de las FAS: un estudio en militares profesionales de tropa y marinería” (approval reference CEI24-015; SEFYCU 341574228; registry output number 11872/2024). The approval was electronically accepted on 19 December 2024. All participants received an information sheet and provided informed consent electronically prior to participation. No IP addresses or other direct identifiers were collected.

Measures. In this study, sex was defined as biological sex as reported by participants in the survey. Job stress was assessed with the Job Stress Survey (JSS), using the perceived intensity of 30 job-related stressors rated from 1 (not stressful) to 9 (extremely stressful) (20). Distributive justice was measured with the distributive justice items from the organisational justice measure (1 to 5 response format) (12). Psychological capital was measured with the OREA questionnaire (1 to 4 response format) covering hope, efficacy, resilience, and optimism (21). Military identity was measured with the Norwegian Professional Identity Scale adapted to the military context (1 to 7 response format) (15). Well-being was assessed with the GHQ-12 using a 4-point response format; items were scored so that higher totals reflect higher well-being by reverse-coding negatively worded items (range 12–48) (18, 22). Job satisfaction was measured with an index (17), and work engagement with the Utrecht Work Engagement Scale (UWES) (19, 23). Occupational health outcomes were operationalised as a reflective latent construct capturing the shared variance across well-being, job satisfaction, and work engagement indicators; all indicators were oriented so that higher values represent better health and functioning. Different response formats and reverse scoring were used as procedural remedies intended to reduce uniform response patterns in self-report responses. For the PLS-SEM analysis, a reduced-indicator measurement model was used to preserve model parsimony while retaining indicators from validated instruments. The reduction procedure was not intended to develop or validate a new short-form scale, nor to replace the original instruments. Rather, it provided an empirical operationalisation of the MOSALPEM constructs within the present sample. The initial measurement model was specified using the available full item pool from each scale. Indicators with standardised loadings below 0.70 were first considered for removal, consistent with common PLS-SEM guidance on indicator reliability (24). Subsequent decisions considered indicator redundancy, collinearity and measurement-model diagnostics, but item removal was constrained by conceptual coherence: retained indicators had to remain theoretically consistent with the construct definition and, where possible, preserve the main content domains represented in the original instruments.

The final reduced-indicator model retained 28 indicators across the study constructs; item codes and loadings are reported in Table 1. To increase transparency, Supplementary Table S1 compares the full-scale operationalisations with the reduced-indicator model. This comparison includes full-scale reliability, retained indicators, reduced-model reliability and convergent validity, and empirical agreement between full and reduced latent scores. Full–reduced score correspondence was high for job stress, psychological capital, distributive justice and occupational health outcomes, and moderate-to-high for military identity. Because military identity showed the lowest correspondence, findings involving this construct were interpreted with particular caution. Overall, the reduced measurement model was treated as a parsimonious model-based operationalisation for this study, not as an independently validated abbreviated instrument.

**Table 1:** Reduced-indicator set used in PLS-SEM.

Construct	Item code	Original item code	Standardised loading	Brief indicator content
PsyCap	PsyCap1	OREA_8_AUT	0.755	Self-efficacy
	PsyCap2	OREA_9_OPT	0.858	Optimism
	PsyCap3	OREA_10_RE	0.798	Resilience
	PsyCap4	OREA_11_ES	0.867	Hope
JS	JS1	JSS_2_PT	0.857	Perceived intensity of job-related stressor
	JS2	JSS_6_FA	0.915	
	JS3	JSS_7_FO	0.914	
	JS4	JSS_15_FO	0.843	
	JS5	JSS_29_FA	0.876	
MI	MI1	IDM_6	0.936	Military professional identity
	MI2	IDM_7	0.873	
	MI3	IDM_11	0.883	
	MI4	IDM_18	0.829	
DJ	DJ1	FJD_1	0.881	Perceived fairness of outcomes
	DJ2	FJD_2	0.913	
	DJ3	FJD_4	0.853	
OHO	OHO1	GHQ_3	0.800	Psychological well-being
	OHO2	GHQ_8	0.758	
	OHO3	GHQ_9	0.758	
	OHO4	GHQ_10	0.783	
	OHO5	JQ_8	0.871	Job satisfaction
	OHO6	JQ_9	0.902	
	OHO7	JQ_12	0.858	
	OHO8	JQ_13	0.908	
	OHO9	JQ_18	0.805	
	OHO10	WES_2	0.811	Work engagement
	OHO11	WES_3	0.859	
	OHO12	WES_7	0.869	

Original item codes identify the retained indicators from the source instruments. Full item wording is not reproduced to respect instrument copyright.

Data analysis. Prior to modelling, data were screened for implausible response patterns, including completion time, attention checks and repetitive responding, and for multivariate outliers using Mahalanobis distance (*p* < 0.001). Fourteen cases (3.7%) met the outlier criterion, but they were retained after quality checks; sensitivity analyses excluding these cases yielded substantively similar estimates. Only complete questionnaires were included; therefore, there were no missing data in the analytic sample. Partial Least Squares Structural Equation Modelling (PLS-SEM) was used to estimate the hypothesised paths because of its suitability for prediction-oriented models with multiple mediators and composite outcome modelling (24, 25). Models were estimated in SmartPLS 4 (SmartPLS GmbH, Oststeinbek, Germany). The measurement model was evaluated following established PLS-SEM criteria. Indicator reliability was assessed using standardised outer loadings, with values of 0.70 or higher considered acceptable and lower-loading indicators examined for possible removal when theoretically justified (24). Internal consistency was evaluated using Cronbach’s alpha and composite reliability, with values above 0.70 considered acceptable and values above 0.95 interpreted cautiously as possible evidence of indicator redundancy. Convergent validity was assessed using average variance extracted (AVE), with values of 0.50 or higher indicating that the construct explained at least half of the variance of its indicators (24). Discriminant validity was examined using the Fornell-Larcker criterion and the HTMT ratio, with HTMT values below 0.90 considered acceptable (26, 27). Collinearity was examined using variance inflation factors (VIF). All inner VIFs were below 3.3 (range 1.00–2.10), indicating no problematic multicollinearity among predictors (28). Potential sources of bias, including self-selection and common method variance, were addressed through procedural remedies, such as anonymity and mixed response formats (29). Structural paths and indirect associations were tested using non-parametric bootstrapping with 10,000 resamples (two-tailed, alpha = 0.05). Effect sizes were interpreted using f-squared (30). Predictive relevance was evaluated using 10-fold cross-validation (PLSpredict-style) comparing PLS-based prediction errors (RMSE and MAE) against linear-model and naive benchmarks. Robustness analyses re-estimated the final structural model with (i) well-being, job satisfaction, and work engagement modelled separately and (ii) available observed covariates (work-life balance measures and sickness absence) included as controls.

## Results

Participant characteristics are summarised in Table 2. Respondents were predominantly men (84.6%) with a mean age of 34.46 years (SD 8.47). Most held temporary contracts (83.2%) and served mainly in the Army (67.5%).

**Table 2:** Participant characteristics.

Characteristic	Value
Sample size	382
Age, years, mean (SD)	34.46 (8.47)
Sex, n (%)	
Women	59 (15.4)
Men	323 (84.6)
Marital status, n (%)	
Married/partnered	258 (67.6)
Single	86 (22.5)
Divorced/separated	38 (9.9)
Children, mean (SD)	1.76 (1.07)
Rank, n (%)	
Soldier/Sailor	213 (55.8)
Corporal	98 (25.7)
Corporal first class	67 (17.5)
Senior corporal	4 (1.0)
Contract type, n (%)	
Temporary	318 (83.2)
Permanent	64 (16.8)
Service branch/destination, n (%)	
Army	258 (67.5)
Navy (shore)	58 (15.2)
Air and Space Force	58 (15.2)
Navy (afloat)	8 (2.1)
Work-life balance measures, n (%)	
Yes	127 (33.3)
No	255 (66.7)
Days in last year, mean (SD)	
Away from base (domestic, paid)	15.04 (37.78)
Away from base (international, paid)	19.42 (76.20)
Sick leave	9.47 (48.85)

To assess the extent to which the sample approximated the target population, its distribution by sex, service branch and rank was descriptively compared with available official data for Spanish professional enlisted military personnel. The sample closely mirrored the population distribution by sex (men: 84.6% vs. 84.7%; women: 15.4% vs. 15.3%), service branch (Army: 67.5% vs. 67.7%; Navy: 17.3% vs. 17.3%; Air and Space Force: 15.2% vs. 15.1%), and rank (senior corporal: 1.0% vs. 1.5%; corporal first class: 17.5% vs. 15.7%; corporal: 25.7% vs. 25.3%; soldier/sailor: 55.8% vs. 57.5%). These comparisons indicate a close descriptive correspondence between the sample and the target population on these key characteristics; however, they should not be interpreted as demonstrating statistical representativeness because recruitment was non-probabilistic.

Descriptive statistics and bivariate correlations are presented in Table 3. Job stress intensity averaged 174.927 (SD = 35.324), and distributive justice averaged 1.414 (SD = 0.556). Job stress correlated negatively with well-being, job satisfaction and work engagement, whereas psychological capital showed strong positive correlations with these outcomes. Job stress also showed a positive zero-order correlation with military identity; this bivariate association was therefore interpreted separately from the adjusted structural path estimated in the PLS-SEM model.

**Table 3:** Descriptive statistics, internal consistency and correlations among study variables.

Variable	Mean (SD)	Alpha	1	2	3	4	5	6	7
1. Well-being (GHQ-12 score)	28.037 (8.612)	0.926	-						
2. Job satisfaction (JQ score)	57.974 (18.555)	0.958	0.872	-					
3. Work engagement (UWES score)	4.397 (1.235)	0.929	0.700	0.872	-				
4. Job stress (JSS intensity)	174.927 (35.324)	0.922	−0.552	−0.512	−0.451	-			
5. Psychological capital (OREA score)	2.831 (0.571)	0.896	0.759	0.774	0.753	−0.488	-		
6. Military identity (NPIS score)	5.055 (0.654)	0.846	0.034	0.224	0.385	0.186	−0.013	-	
7. Distributive justice (Colquitt DJ)	1.414 (0.556)	0.872	0.257	0.368	0.267	−0.109	0.251	0.142	-

Correlations are Pearson coefficients. Alpha = Cronbach’s alpha. Higher GHQ-12 scores indicate better well-being, that is, less distress, after reverse scoring.

Measurement model evaluation supported adequate reliability, convergent validity and discriminant validity. The final reduced-indicator measurement model comprised 28 indicators across five constructs (Table 1), with standardised loadings ranging from 0.755 to 0.936, composite reliabilities from 0.892 to 0.964, Cronbach’s alpha values from 0.837 to 0.960, and AVE values from 0.674 to 0.779. These values met the criteria described in the data analysis section. Discriminant validity was supported by the Fornell-Larcker criterion and HTMT ratios, all of which were ≤ 0.856.

Supplementary Table S1 provides an additional comparison between the full-scale operationalisations and the reduced-indicator measurement model. Full–reduced latent score correlations ranged from 0.876 to 0.987, with military identity showing the lowest correspondence (*r* = 0.876). An additional sensitivity analysis therefore re-estimated the structural model using the full 33-item NPIS. Although the point estimates retained the same direction, bootstrap uncertainty increased substantially for paths involving military identity. Nevertheless, explained variance in OHO remained highly similar (R^2^ = 0.724 versus 0.735), and the total association between JS and OHO was virtually unchanged (*β* = −0.705 versus −0.707). Detailed results are provided in Supplementary Table S5.

Structural model results are shown in Table 4. JS was negatively associated with PsyCap (*β* = −0.683), DJ (*β* = −0.351), MI (*β* = −0.331) and OHO (*β* = −0.197). DJ was positively associated with MI (*β* = 0.164). OHO was positively associated with PsyCap (*β* = 0.579) and MI (*β* = 0.295). All structural paths were statistically significant, and the bootstrapped 95% confidence intervals did not include zero. Regarding model explanatory power, R^2^ values for the endogenous constructs were as follows: PsyCap = 0.466, DJ = 0.123, MI = 0.174 and OHO = 0.735. Expressed as explained variance, the model accounted for 46.6% of PsyCap, 12.3% of DJ, 17.4% of MI and 73.5% of OHO.

**Table 4:** Structural model bootstrapping estimation.

Path	β	t-stat	*p*-value	95% CI	f^2^
JS → OHO	−0.197	3.912	<0.001	[−0.290, −0.093]	0.069
JS → DJ	−0.351	9.312	<0.001	[−0.428, −0.281]	0.140
JS → PsyCap	−0.683	29.809	<0.001	[−0.727, −0.637]	0.873
JS → MI	−0.331	7.815	<0.001	[−0.415, −0.250]	0.116
DJ → MI	0.164	5.263	<0.001	[0.101, 0.223]	0.029
MI → OHO	0.295	10.716	<0.001	[0.244, 0.353]	0.279
PsyCap → OHO	0.579	14.261	<0.001	[0.502, 0.661]	0.671

Indirect and total effects are summarised in Table 5. JS was indirectly associated with OHO mainly through PsyCap (βind = −0.395, 95% CI [−0.467, −0.331]) and, to a lesser extent, through MI (βind = −0.098, 95% CI [−0.136, −0.067]). A smaller sequential indirect association was observed through DJ and MI (βind = −0.017, 95% CI [−0.024, −0.011]). The sum of the three indirect associations was −0.510. Together with the direct association between JS and OHO (*β* = −0.197), this yielded a total association of *β* = −0.707.

**Table 5:** Indirect and total effects of job stress on occupational health outcomes.

Effect	β	t-stat	*p*-value	95% CI
Indirect effects				
JS → PsyCap → OHO	−0.395	11.340	<0.001	[−0.467, −0.331]
JS → MI → OHO	−0.098	5.518	<0.001	[−0.136, −0.067]
JS → DJ → MI → OHO	−0.017	4.856	<0.001	[−0.024, −0.011]
Total effects				
JS - > OHO	−0.707	27.083	<0.001	[−0.756, −0.655]

Confidence intervals are percentile bootstrap intervals based on 10,000 resamples.

Effect sizes indicated a large association between JS and PsyCap (f^2^ = 0.873), and between PsyCap and OHO (f^2^ = 0.671). MI showed a medium association with OHO (f^2^ = 0.279), whereas the remaining effects were small.

Robustness analyses modelling well-being, job satisfaction and work engagement separately yielded consistent patterns. JS was negatively associated with well-being and job satisfaction, whereas its direct association with work engagement was small and included zero. PsyCap remained positively associated with all three outcomes, and MI was most strongly associated with job satisfaction and work engagement. This pattern supports the broader occupational health outcomes operationalisation, while also indicating that work engagement may capture a comparatively more motivational dimension that is less directly associated with job stress once psychological capital and military identity are included in the model. A covariate-adjusted model including binary indicators for use of any work-life balance measure and for more than 7 days of sickness absence in the previous year produced substantively similar estimates: covariate effects were negligible and the main path coefficients changed minimally. Detailed robustness results are provided in Supplementary Tables S2, S3.

Predictive assessment showed positive cross-validated predictive relevance for all endogenous constructs, with Q^2^ values of 0.114 for DJ, 0.458 for PsyCap, 0.139 for MI and 0.485 for OHO. For OHO, PLS-based predictions showed lower overall prediction error than the naive benchmark, but higher error than the linear-model benchmark. At the indicator level, PLS-based predictions reduced error relative to the naive benchmark for all 12 indicators; however, the linear-model benchmark yielded lower errors, as shown in Supplementary Table S4.

The higher-order operationalisation of occupational health outcomes was theoretically driven and aimed at capturing the shared variance among positive occupational functioning indicators. Psychological well-being, job satisfaction and work engagement were therefore modelled as related but non-redundant dimensions representing the broader occupational health outcomes construct. However, the robustness analyses also showed that these dimensions should not be treated as fully interchangeable. In particular, the direct association between job stress and work engagement was small and not statistically different from zero when work engagement was modelled separately, whereas the associations with well-being and job satisfaction remained negative. This suggests that the higher-order construct is useful for representing a general pattern of occupational functioning, but that work engagement may be more strongly linked to motivational and resource-related pathways than to the direct association with job stress.

## Discussion

This study provides findings consistent with MOSALPEM within a military occupational context and contributes to the growing literature on psychosocial correlates of occupational health in high-demand work environments. Consistent with Conservation of Resources (COR) theory, job stress was strongly associated with reduced psychological capital, suggesting that chronic occupational demands may be linked to lower levels of the personal resources individuals rely upon to cope with adversity and maintain positive functioning (3, 13, 14). The magnitude of this association highlights the relevance of stress within resource-loss explanations and is consistent with the proposition that occupational health is associated not only with exposure to demands but also with the availability of psychosocial resources.

The findings are also broadly consistent with the Job Demands–Resources (JD-R) model. Psychological capital and military identity were positively associated with occupational health outcomes, suggesting that both personal and identity-related resources may be relevant to employees’ reported well-being, satisfaction and engagement despite exposure to occupational stressors (4–6, 31). Psychological capital emerged as the strongest direct predictor of occupational health outcomes within the model. This finding is consistent with previous evidence identifying hope, efficacy, resilience and optimism as psychological resources associated with adaptive functioning and lower vulnerability to stress-related outcomes (13, 14). From a resource-based perspective, this pattern is consistent with psychological capital as a personal resource associated with motivation and positive evaluations of the work environment.

A particularly relevant contribution concerns the role of military identity. Although identity-related processes have received increasing attention in organisational research, they remain comparatively underrepresented in occupational health models. In the present study, military identity demonstrated a positive association with occupational health outcomes, a pattern consistent with the interpretation of military identity as an identity-related psychosocial resource. Personnel who perceive military service as a central component of their self-concept may report greater meaning, purpose and social support from their occupational role, which may be associated with more positive occupational functioning even under demanding conditions. This interpretation is consistent with social identity perspectives, which propose that group identification can provide psychological resources that facilitate coping, resilience and collective efficacy.

The relationship between job stress and military identity requires particular caution. As shown in the bivariate correlations, job stress and military identity were positively correlated at the zero-order level, whereas the adjusted structural path from job stress to military identity was negative. This change in sign should not be interpreted as a contradiction or as evidence that job stress reduces military identity over time. Rather, it indicates that the bivariate and structural coefficients answer different questions. The positive zero-order correlation suggests that, in this sample, personnel reporting higher stress also tended to report stronger identification with the military role. This interpretation is also compatible with organisational identification research showing that identification may be associated with employee well-being through social support and collective efficacy (16), further highlighting the importance of distinguishing zero-order associations from adjusted structural relationships. This pattern is plausible in a military context, where stronger identification may coexist with greater exposure to demanding duties, greater involvement in the organisation or greater salience of military role expectations. By contrast, the negative structural path reflects the conditional association between job stress and military identity within the multivariable latent-variable model, after accounting for the broader system of psychosocial resources represented in MOSALPEM. The sign reversal is therefore compatible with a suppression or adjustment effect arising from shared variance among model constructs and from the distinction between bivariate correlations and adjusted latent paths. Given the cross-sectional and self-report design, this finding should be interpreted as a conditional association rather than as a causal process, and future longitudinal research should examine whether job stress and military identity show different relationships across levels of organisational fairness, psychological resources and occupational involvement.

The results also highlight the importance of distributive justice within military organisations. The negative association between job stress and distributive justice aligns with theoretical and empirical evidence suggesting that demanding work environments may be associated with stronger perceptions of inequity, particularly when employees perceive an imbalance between their efforts and the rewards, recognition or opportunities they receive (7, 9–12). Distributive justice was also positively associated with military identity, suggesting that fairness perceptions may be linked to employees’ sense of belonging and institutional commitment. This is theoretically relevant because organisational resources may be linked to occupational health not only directly but also indirectly through identity-related pathways. In highly hierarchical organisations such as the armed forces, perceptions of fairness may therefore be relevant to positive employee attitudes and identification with the institution. More broadly, research in organisational settings has shown that relational resources may be associated with organisational outcomes through distinct intermediate mechanisms, including knowledge-sharing and knowledge-acquisition pathways (32). This further supports the value of examining indirect pathways when modelling complex organisational processes.

Taken together, the findings are consistent with the central proposition underlying MOSALPEM: occupational health in military work environments is associated with an interconnected system of demands, personal resources, organisational resources and identity-related processes. Job stress was not only directly associated with occupational health outcomes, but also showed indirect associations through lower levels of psychosocial resources. This pattern may be consistent with resource-loss explanations described by COR theory and highlights resource preservation and resource development as relevant targets for future longitudinal and intervention research in military contexts.

From a theoretical perspective, MOSALPEM should not be understood as an alternative to established occupational health frameworks such as COR theory or the JD-R model. Rather, the model can be viewed as a context-specific extension that integrates personal, organisational and identity-related resources within a single explanatory framework tailored to military work environments. While COR theory and JD-R theory provide robust explanations for the role of demands and resources in employee well-being, the present findings suggest that identity-related mechanisms may warrant greater attention in occupational settings characterised by strong institutional cultures, formal hierarchies and collective role expectations. In this regard, the inclusion of military identity represents one of the main conceptual contributions of the model.

The practical implications of these findings are also important. Occupational health interventions in military organisations have traditionally focused on reducing stress exposure and preventing psychological disorders. Although these objectives remain essential, the present findings suggest that occupational health promotion in military settings may need to consider a broader resource-based framework. Future longitudinal and intervention studies should examine whether reducing excessive workload, chronic time pressure and role ambiguity is associated with lower resource depletion, and whether initiatives aimed at strengthening psychological capital are associated with better coping with occupational demands. Similarly, organisational practices related to transparency, fairness, recognition and equitable access to opportunities may be associated with stronger military identity and more positive occupational experiences. Leadership development programmes may be particularly relevant to examine in this regard, given their potential association with both justice perceptions and organisational identification.

The potential relevance of MOSALPEM may extend beyond the military domain. Although the present study focused on Spanish enlisted personnel, the psychosocial association patterns observed here may be relevant to other highly structured occupational systems, including policing, correctional services, emergency response organisations and private security sectors. Future research should examine whether similar resource-based association patterns are observed across different institutional contexts and whether identity-related resources show comparable associations with occupational health outcomes.

Several limitations should be acknowledged. The cross-sectional design precludes strong causal inferences and does not allow the temporal ordering of resource-loss processes to be directly examined. The use of self-report measures introduces the possibility of common method variance and social desirability effects, although procedural remedies were implemented and diagnostic indicators suggested that common method bias was unlikely to account for the observed relationships (28, 29). Recruitment through professional military associations may also have introduced self-selection effects and prevented the calculation of a precise response rate. In addition, the non-probabilistic recruitment strategy and the relatively low estimated minimum response rate limit the representativeness of the sample. Although the sample distribution on selected characteristics was compared with available population benchmarks, these similarities do not establish statistical representativeness. The findings should therefore be generalised cautiously even within the Spanish Armed Forces and should not be assumed to apply directly to other national military systems or non-military occupational settings. Moreover, well-being may also be associated with non-occupational factors not incorporated into MOSALPEM, including family composition and parenthood, which have been associated with differences in self-reported happiness (33). Furthermore, the occupational health outcomes construct aggregated indicators of psychological well-being, job satisfaction and work engagement. Although this approach was theoretically justified and facilitated model parsimony, it may conceal differential associations across individual dimensions of occupational health.

This issue was partly addressed through robustness analyses in which psychological well-being, job satisfaction and work engagement were modelled separately. These analyses supported the general pattern observed in the main model, as psychological capital remained positively associated with all three outcomes and military identity was especially relevant to job satisfaction and work engagement. Nevertheless, the direct association between job stress and work engagement was small and not statistically different from zero. This finding suggests that engagement should not be interpreted simply as the inverse of job stress or as an outcome associated with stress in the same way as well-being and job satisfaction. Rather, engagement may represent a more motivational component of occupational functioning, potentially linked more strongly to personal and identity-related resources than to job stress itself. Accordingly, the higher-order occupational health outcomes construct should be interpreted as a parsimonious representation of shared positive occupational functioning, while recognising that its individual dimensions may show partly distinct associations.

Future longitudinal and multi-source studies should examine these dimensions separately and jointly to determine whether the MOSALPEM pathways operate similarly across well-being, job satisfaction and work engagement. Studies incorporating objective indicators such as absenteeism, healthcare utilisation, retention intentions and administrative personnel records would strengthen causal inference and provide a more comprehensive evaluation of the mechanisms proposed by MOSALPEM.

In conclusion, the present findings provide evidence that occupational health among military personnel is associated not only with exposure to occupational stressors but also with the availability of personal, organisational and identity-related resources. By integrating these elements within a single framework, MOSALPEM offers a theoretically grounded approach for examining occupational health in military work environments and provides a foundation for future longitudinal and intervention research on employee well-being and organisational effectiveness.

## Conclusion

This study examined psychosocial associations linking job stress to occupational health outcomes among enlisted personnel in the Spanish Armed Forces and provided findings consistent with the Psychosocial Occupational Health Model for Military Work Environments (MOSALPEM). The findings indicate that job stress was associated with poorer occupational health outcomes both directly and indirectly through lower levels of key psychosocial resources, particularly psychological capital and military identity. In addition, distributive justice was indirectly associated with occupational health through military identity, highlighting the relevance of fairness perceptions within highly hierarchical organisational systems.

These findings are consistent with the central proposition of MOSALPEM that occupational health in military work environments is associated not only with exposure to occupational demands but also with the availability and preservation of personal, organisational and identity-related resources. The substantial proportion of variance explained in occupational health outcomes (73.5%) suggests that the model captured important psychosocial patterns of association involving employee well-being, job satisfaction and work engagement in military contexts.

From a theoretical perspective, the study contributes to the occupational health literature by integrating resource-based explanations derived from Conservation of Resources theory and the Job Demands–Resources model within a framework specifically adapted to military organisations. In particular, the findings highlight the potential relevance of military identity as a psychosocial resource that complements more traditional personal and organisational resources in accounting for occupational health outcomes. This contribution may be especially relevant for occupational settings characterised by strong institutional cultures, formal hierarchies, and collective role expectations.

From a practical standpoint, the results suggest that occupational health promotion within military organisations should extend beyond the reduction of psychosocial stressors alone. Future intervention studies should evaluate combined approaches aimed at reducing excessive demands, enhancing perceptions of distributive fairness, strengthening organisational trust and recognition, and developing psychological capital through targeted training and support programmes. Longitudinal and intervention studies should examine whether such initiatives are associated not only with improved employee well-being but also with greater organisational effectiveness, commitment and operational readiness.

Although the present study focused on Spanish enlisted personnel, the association patterns observed may have broader relevance for other highly structured and disciplined occupational environments. Future longitudinal and multi-source studies should examine the temporal dynamics proposed by MOSALPEM, evaluate causal pathways more rigorously, and determine whether similar resource-based association patterns are observed across different military and non-military contexts. Continued research in this area may contribute to the development of more comprehensive occupational health models for examining the unique challenges associated with demanding institutional environments.

## Data Availability

The raw data supporting the conclusions of this article will be made available by the authors, without undue reservation.
