# Supplementary material for: Psychosocial occupational health in military work environments: cross-sectional findings among Spanish enlisted military personnel
Source: Front Public Health. 2026 Oct 8;14:1996254. doi: 10.3389/fpubh.2026.1996254 (PMC13686988; doi:10.3389/fpubh.2026.1996254)
Supplement: Supplementary file 1 [file Table_1.docx]

**STROBE Statement—Checklist for cross-sectional studies**

**TITLE AND ABSTRACT**

| **Item** | **Recommendation** | **Reported (Page/Section)** |
| --- | --- | --- |
| 1a | Indicate study design in title/abstract | Abstract |
| 1b | Informative and balanced summary | Abstract |

**INTRODUCTION**

| **Item** | **Recommendation** | **Reported** |
| --- | --- | --- |
| 2 | Background/rationale | Introduction |
| 3 | Objectives/hypotheses | Introduction |

**METHODS**

| Item | Recommendation | Reported |
| --- | --- | --- |
| 4 | Study design | Methods |
| 5 | Setting | Methods |
| 6a | Participants eligibility | Methods |
| 7 | Variables clearly defined | Methods |
| 8 | Data sources/measurement | Methods |
| 9 | Bias | Methods + Discussion |
| 10 | Study size | Methods |
| 11 | Quantitative variables | Methods |
| 12a | Statistical methods | Methods |
| 12b | Subgroups/interactions | Results |
| 12c | Missing data | Methods |
| 12d | Sampling strategy | Methods |
| 12e | Sensitivity analyses | Methods |

**RESULTS**

| Item | Recommendation | Reported |
| --- | --- | --- |
| 13a | Participants flow | Methods/Results |
| 13b | Reasons for non-participation | Methods |
| 13c | Flow diagram | Not included |
| 14a | Descriptive data | Results |
| 14b | Missing data | Methods |
| 15 | Outcome data | Results |
| 16 | Main results | Results |
| 17 | Other analyses | Results |

**DISCUSSION**

| Item | Recommendation | Reported |
| --- | --- | --- |
| 18 | Key results | Discussion |
| 19 | Limitations | Discussion |
| 20 | Interpretation | Discussion |
| 21 | Generalizability | Discussion |

**OTHER INFORMATION**

| Item | Recommendation | Reported |
| --- | --- | --- |
| 22 | Funding | Funding details section |

**Supplementary Table S1. Traceability and measurement evidence for the reduced-indicator set in PLS-SEM**

| Construct | Full-scale operationalisation | Full-scale reliability | Retained original indicators | Manuscript indicator codes | Reduced model evidence | Full–reduced score equivalence |
| --- | --- | --- | --- | --- | --- | --- |
| Job stress (JS) | Job Stress Survey intensity score; 30 job-related stressors (JSS_1–JSS_30) covering work pressure, lack of support, lack of organisation and lack of professional fulfilment. | α = 0.922 | JSS_2_PT, JSS_6_FA, JSS_7_FO, JSS_15_FO, JSS_29_FA | JS1–JS5 | Loadings = 0.843–0.915;  α = 0.928;  CR = 0.946;  AVE = 0.777 | r = 0.960, 95% CI [0.951, 0.967];  MAE = 0.244;  RMSE = 0.283;  mean Δ = 0.000 |
| Psychological capital (PsyCap) | OREA questionnaire; 12 items representing optimism, resilience, hope and self-efficacy. | α = 0.896 | OREA_8_AUT, OREA_9_OPT, OREA_10_RE, OREA_11_ES | PsyCap1–PsyCap4 | Loadings = 0.755–0.867;  α = 0.837;  CR = 0.892;  AVE = 0.674 | r = 0.928, 95% CI [0.912, 0.941];  MAE = 0.321;  RMSE = 0.380;  mean Δ = 0.000 |
| Military identity (MI) | Adapted Norwegian Professional Identity Scale; 33 items (NPIS_1–NPIS_33) representing professional, idealistic and individualistic dimensions of military identity. | α = 0.846 | NPIS_6, NPIS_7, NPIS_11, NPIS_18 | MI1–MI4 | Loadings = 0.829–0.936;  α = 0.905;  CR = 0.933;  AVE = 0.777 | r = 0.876, 95% CI [0.851, 0.898];  MAE = 0.370;  RMSE = 0.497;  mean Δ = 0.000 |
| Distributive justice (DJ) | Distributive justice factor from the organisational justice scale; four items (FJD_1–FJD_4) assessing perceived fairness of outcomes. | α = 0.872 | FJD_1,  FJD_2, FJD_4 | DJ1–DJ3 | Loadings = 0.853–0.913;  α = 0.859;  CR = 0.914;  AVE = 0.779 | r = 0.987, 95% CI [0.984, 0.989];  MAE = 0.105;  RMSE = 0.162;  mean Δ = 0.000 |
| Occupational health outcomes (OHO) | Higher-order occupational health outcome operationalisation based on well-being, job satisfaction and work engagement indicators. The full operationalisation included GHQ-12, JQ and UWES indicators. | GHQ-12  α = 0.926;  JQ  α = 0.958;  UWES  α = 0.929 | GHQ_3, GHQ_8, GHQ_9, GHQ_10,  JQ_8,  JQ_9,  JQ_12,  JQ_13,  JQ_18,  WES_2, WES_3,  WES_7 | OHO1–OHO12 | Loadings = 0.758–0.908;  α = 0.960;  CR = 0.964;  AVE = 0.694 | r = 0.980, 95% CI [0.976, 0.984];  MAE = 0.168;  RMSE = 0.200;  mean Δ = 0.000 |

Note: CR = composite reliability; AVE = average variance extracted; MAE = mean absolute error; RMSE = root mean squared error; Δ = reduced latent score minus full latent score. Original item wording is not reproduced to respect instrument copyright.

**Supplementary Table S2. Robustness analyses with outcomes modeled separately**

| **Outcome** | **Path** | **β** | **95% CI** |
| --- | --- | --- | --- |
| Well-being (GHQ-12) | JS → Well-being | -0.233 | [-0.347, -0.123] |
| Well-being (GHQ-12) | PsyCap → Well-being | 0.542 | [0.461, 0.626] |
| Well-being (GHQ-12) | MI → Well-being | 0.144 | [0.086, 0.204] |
| Job satisfaction | JS → Job satisfaction | -0.236 | [-0.338, -0.133] |
| Job satisfaction | PsyCap → Job satisfaction | 0.394 | [0.308, 0.471] |
| Job satisfaction | MI → Job satisfaction | 0.466 | [0.402, 0.524] |
| Work engagement (UWES) | JS → Engagement | -0.062 | [-0.166, 0.054] |
| Work engagement (UWES) | PsyCap → Engagement | 0.539 | [0.442, 0.634] |
| Work engagement (UWES) | MI → Engagement | 0.474 | [0.419, 0.532] |

Note: Confidence intervals are percentile bootstrap intervals based on 10,000 resamples for each model. The direct JS → work engagement path includes zero, indicating attenuation of the direct association when work engagement is modelled alone.

**Supplementary Table S3. Covariate-adjusted model: paths to occupational health outcomes**

| **Path** | **β** | **95% CI** |
| --- | --- | --- |
| Absence → OHO | 0.011 | [-0.043, 0.065] |
| PsyCap → OHO | 0.584 | [0.511, 0.669] |
| WLB → OHO | -0.005 | [-0.057, 0.050] |
| JS → OHO | -0.181 | [-0.272, -0.079] |
| MI → OHO | 0.309 | [0.254, 0.369] |

Note: WLB = binary indicator of using any work-life balance measure; Absence = binary indicator of more than seven days of sickness absence in the previous year. Confidence intervals are percentile bootstrap intervals based on 10,000 resamples.

**Supplementary Table S4. PLSpredict-style indicator-level prediction errors for occupational health outcomes**

| **Indicator** | **RMSE_PLS** | **RMSE_LM** | **RMSE_Naive** | **MAE_PLS** | **MAE_LM** | **MAE_Naive** |
| --- | --- | --- | --- | --- | --- | --- |
| OHO1 | 0.913 | 0.823 | 1.005 | 0.685 | 0.629 | 0.877 |
| OHO2 | 0.815 | 0.655 | 1.003 | 0.643 | 0.506 | 0.853 |
| OHO3 | 0.850 | 0.830 | 1.004 | 0.705 | 0.667 | 0.918 |
| OHO4 | 0.809 | 0.770 | 1.002 | 0.663 | 0.589 | 0.894 |
| OHO5 | 0.811 | 0.708 | 1.005 | 0.640 | 0.535 | 0.896 |
| OHO6 | 0.802 | 0.598 | 1.003 | 0.635 | 0.457 | 0.891 |
| OHO7 | 0.752 | 0.638 | 1.002 | 0.620 | 0.505 | 0.921 |
| OHO8 | 0.802 | 0.657 | 1.006 | 0.628 | 0.494 | 0.877 |
| OHO9 | 0.923 | 0.792 | 1.003 | 0.725 | 0.615 | 0.863 |
| OHO10 | 0.812 | 0.619 | 1.003 | 0.587 | 0.439 | 0.850 |
| OHO11 | 0.737 | 0.571 | 1.005 | 0.588 | 0.428 | 0.871 |
| OHO12 | 0.815 | 0.677 | 1.007 | 0.632 | 0.521 | 0.885 |

Note: OHO item codes correspond to the retained occupational health outcome indicators reported in Table I. Indicators were standardised using training-set scaling in each fold.

**Supplementary Table S5. Sensitivity analysis using the full 33-item NPIS for military identity**

| **Effect** | **Reduced-indicator model β** | **95% CI** | **p** | **Full 33-item NPIS β** | **95% CI** | **p** |
| --- | --- | --- | --- | --- | --- | --- |
| **Structural paths** |  |  |  |  |  |  |
| JS → PsyCap | −0.683 | [−0.727, −0.637] | <0.001 | −0.682 | [−0.726, −0.635] | <0.001 |
| JS → DJ | −0.351 | [−0.428, −0.281] | <0.001 | −0.348 | [−0.426, −0.278] | <0.001 |
| JS → MI | −0.331 | [−0.415, −0.250] | <0.001 | −0.569 | [−0.644, 0.657] | 0.137 |
| JS → OHO | −0.197 | [−0.290, −0.093] | <0.001 | −0.156 | [−0.240, −0.053] | 0.001 |
| DJ → MI | 0.164 | [0.101, 0.223] | <0.001 | 0.243 | [−0.242, 0.337] | 0.103 |
| MI → OHO | 0.295 | [0.244, 0.353] | <0.001 | 0.333 | [−0.359, 0.387] | 0.121 |
| PsyCap → OHO | 0.579 | [0.502, 0.661] | <0.001 | 0.485 | [0.426, 0.549] | <0.001 |
| **Specific indirect effects** |  |  |  |  |  |  |
| JS → PsyCap → OHO | −0.395 | [−0.467, −0.331] | <0.001 | −0.331 | [−0.386, −0.281] | <0.001 |
| JS → MI → OHO | −0.098 | [−0.136, −0.067] | <0.001 | −0.189 | [−0.240, −0.155] | <0.001 |
| JS → DJ → MI → OHO | −0.017 | [−0.024, −0.011] | <0.001 | −0.028 | [−0.040, −0.016] | <0.001 |
| Total effect |  |  |  |  |  |  |
| JS → OHO | −0.707 | [−0.756, −0.655] | <0.001 | −0.705 | [−0.754, −0.652] | <0.001 |
| **Explained variance** |  |  |  |  |  |  |
| R² MI | 0.174 | — | — | 0.479 | — | — |
| R² OHO | 0.735 | — | — | 0.724 | — | — |

Note: JS = job stress; PsyCap = psychological capital; DJ = distributive justice; MI = military identity; OHO = occupational health outcomes. The reduced-indicator model operationalised MI using four retained NPIS indicators, whereas the sensitivity model used all 33 NPIS items. Bootstrap inference was based on 10,000 subsamples, two-tailed testing, α = 0.05, and percentile 95% confidence intervals. All other measurement and structural specifications were held constant.
